# Protein A-Mediated Binding of *Staphylococcus* spp. to Antibodies in Flow Cytometric Assays and Reduction of This Binding by Using Fc Receptor Blocking Reagent

**DOI:** 10.1128/AEM.01435-20

**Published:** 2020-08-18

**Authors:** Ultan P. Cronin, Laura Girardeaux, Elaine O’Meara, Martin G. Wilkinson

**Affiliations:** a Department of Biological Sciences, University of Limerick, Castletroy, Ireland; b LEGTPA Bordeaux Blanquefort (Lycée Agro-Viticole de Blanquefort), Blanquefort, Bordeaux, France; The Pennsylvania State University

**Keywords:** protein A, *Staphylococcus*, antibodies, assay development, flow cytometry, microbial detection

## Abstract

Staphylococcus aureus and other coagulase-positive *Staphylococcus* spp. bind the Fc region of IgG antibodies through expression of protein A (SpA). These species have consequently been a source of false-positive signals in antibody-based assays designed to detect other target bacteria. Here, flow cytometry was used to study the influence of a number of factors on the SpA-mediated binding of single cells to an anti-human IgG antibody, including strain, heat killing, overnight storage, growth phase, cell physiology, surface adhesion, and growth in model food systems. Through the costaining of antibody-stained cells with the permeability dye propidium iodide and calcein violet AM, the cell physiological status was related to SpA-mediated antibody binding. Generally, permeabilized cells lacking esterase activity did not strongly bind antibody. The binding of a number of commercially available polyclonal IgG antibodies to non-*Staphylococcus* spp. was also characterized. Not all SpA-expressing species showed strong binding of mouse IgG, and one species not known to express SpA showed strong binding. Most SpA-expressing strains bound rabbit IgG antibodies to some extent, whereas only one strain bound goat IgG. To reduce or eliminate SpA-mediated IgG binding, the following products were evaluated as blocking reagents and applied prior to staining with primary or secondary antibody: normal rabbit serum, mouse IgG isotype control, goat IgG, and a commercial FcR blocking reagent. Only the FcR blocking reagent consistently reduced SpA-mediated binding of *Staphylococcus* spp. to antibodies against other species and could be recommended as a blocking reagent in immunoassays designed to detect non-*Staphylococcus* species.

**IMPORTANCE** This study characterizes a widespread but little-studied problem associated with the antibody-based detection of microbes—the *Staphylococcus* protein A (SpA)-mediated binding of IgG antibodies—and offers a solution: the use of commercial FcR blocking reagent. A common source of false-positive signals in the detection of microbes in clinical, food, or environmental samples can be eliminated by applying this study’s findings. Using flow cytometry, the authors demonstrate the extent of heterogeneity in a culture’s SpA-mediated binding of antibodies and that the degree of SpA-mediated antibody binding is strain, growth phase, and food matrix dependent and influenced by simulated food processing treatments and cell adherence. In addition, our studies of SpA-mediated binding of *Staphylococcus* spp. to antibodies against other bacterial species produced a very nuanced picture, leading us to recommend testing against multiple strains of S. aureus and *S. hyicus* of all antibodies to be incorporated into any immunoassay designed to detect a non-*Staphylococcus* spp.

## Abstract (precis)

This study characterizes a widespread but little-studied problem associated with the antibody-based detection of microbes—the *Staphylococcus* protein A (SpA)-mediated binding of IgG antibodies—and offers a solution: the use of commercial FcR blocking reagent. A common source of false-positive signals in the detection of microbes in clinical, food, or environmental samples can be eliminated by applying this study’s findings. Using flow cytometry, the authors demonstrate the extent of heterogeneity in a culture’s SpA-mediated binding of antibodies and that the degree of SpA-mediated antibody binding is strain, growth phase, and food matrix dependent and influenced by simulated food processing treatments and cell adherence. In addition, our studies of SpA-mediated binding of *Staphylococcus* spp. to antibodies against other bacterial species produced a very nuanced picture, leading us to recommend testing against multiple strains of S. aureus and *S. hyicus* of all antibodies to be incorporated into any immunoassay designed to detect a non-*Staphylococcus* spp.

## INTRODUCTION

Researchers developing antibody-based detection methods for bacteria are familiar with the problematic nature of Staphylococcus aureus as the origin of a high proportion of false-positive signals in many immunoassays. From the earliest days of microbial flow cytometry (FCM), the phenomenon of S. aureus reacting with antibodies raised against other species, and so interfering with the specificity of an assay, has been reported. Other workers, in describing the development of a cytometry-based detection method for Listeria monocytogenes, reported that S. aureus gave rise to high degrees of cross-reactivity, with enrichment for the target organism using selective broth prior to antibody staining being required to overcome this obstacle (1). In reporting an evaluation of a monoclonal antibody’s ability to detect live L. monocytogenes and *L. innocua*, it was found that three of four S. aureus strains tested showed cross-reactivity with the antibody (2). During the development of a flow cytometric method for detection of Escherichia coli O157:H7 in spinach, while not mentioning S. aureus in the context of nonspecificity, Buzatu et al. did refer to possible cross-reactivity with the antibody by the foodstuff’s natural microflora, where S. aureus may be found (3). Cross-reactivity from S. aureus to both a primary antibody against Burkholderia cepacia and an R-phycoerythrin-conjugated secondary monoclonal antibody were encountered (4). The problem of cross-reactivity is not limited to the field of bacterial detection or FCM. It was reported that cross-reactivity with ELISA antibodies for murine interleukin-1β and tumor necrosis factor alpha assays was caused by clinical isolates and laboratory strains of S. aureus, and the authors who conducted that study concluded that data from all such assays should be revalidated (5).

Cross-reactivity from S. aureus and other coagulase-positive *Staphylococcus* spp. arises from the fact that >90% of S. aureus strains express protein A (SpA) on their outer surfaces; this protein shows a high degree of binding to immunoglobulins, especially immunoglobulin G (IgG) (6–8). SpA is present in abundance on the cell wall of S. aureus (up to 7% of total proteins), amounting to ∼80,000 IgG binding sites per cell. This protein contains four or five immunoglobulin-binding domains that bind the Fc region of IgG antibodies and the Fab of variable heavy 3 (VH3) idiotype antibodies (9). Hence, using IgG antibodies against a specific non-*Staphylococcus* target within a suspension of multiple species, including S. aureus and/or other coagulase-positive species (which would describe many food, clinical, or environmental samples), both target cells and S. aureus will contribute signal during the measurement of binding. In certain circumstances, the “true” signal will be swamped by a false-positive signal. Because of SpA’s binding to the VH3 region, unless an antibody is very well characterized, choosing to use non-IgG antibodies does not ensure that a given antibody will not bind to SpA. Indeed, ∼50% of a panel of human IgM and 32 to 54% of human peripheral blood B cells were reported to interact with SpA through the Fab domain (10).

However, some researchers do not attribute a particular cross-reactivity to S. aureus over that of other nontarget species. One group (11) noted that the reaction to a primary anti-*Listeria* mouse IgG antibody by S. aureus FDA209P was low compared to the target, while another group (12) reported that S. aureus ATCC 6538P and ATCC 27217 and S. epidermidis ATCC 12228 did not cross-react with fluorescein isothiocyanate-labeled anti-Escherichia coli O157 IgG monoclonal antibody. These findings may reflect strain-related expression of SpA (13) or that the particular subclass of IgG used did not react strongly with SpA. It was shown that different classes of IgG bind more or less tightly to SpA (14).

This study originated from our experience of S. aureus cross-reacting with antibodies intended to specifically bind to other bacterial species within immuno-FCM-based detection assays. We first wanted to gain an understanding of the phenomenon and so, randomly choosing an anti-human IgG2a antibody generated in mice, we tested the ability of various *Staphylococcus* strains to bind this. In a previous study, we demonstrated that the physiological state and types of treatments to induce cellular damage or death affect the extent of antibody binding of S. aureus (15). In the present study, we investigated the effect of heat killing on the SpA-mediated binding of antibody to S. aureus. Further to this, we studied the effect of storage of *Staphylococcus* strains in water or phosphate-buffered saline (PBS), the influence of the growth phase, and the effects of various simulated food-processing treatments on antibody binding. We then examined *Staphylococcus* SpA-mediated binding of antibody under simulated real-world conditions: cultures spiked onto food-grade stainless-steel surfaces and into model food systems. Using commercial antibodies against a number of non-*Staphylococcus* bacteria and purified serum IgG, we demonstrated the extent of SpA-mediated binding of these antibodies in FCM detection assays. Finally, approaches to block unwanted SpA binding were undertaken using normal rabbit serum, mouse IgG, goat IgG, and FcR blocking reagent. Of these, FcR blocker yielded the most promising results. For a number of *Staphylococcus* spp., SpA-mediated binding to commercial anti-*Enterococcus* and anti-*Bacillus* antibodies was eliminated or reduced by incubation with this product prior to antibody staining.

## RESULTS

### *Staphylococcus* binding of mouse anti-human CD44 antibody is variable.

**FIG 1:**
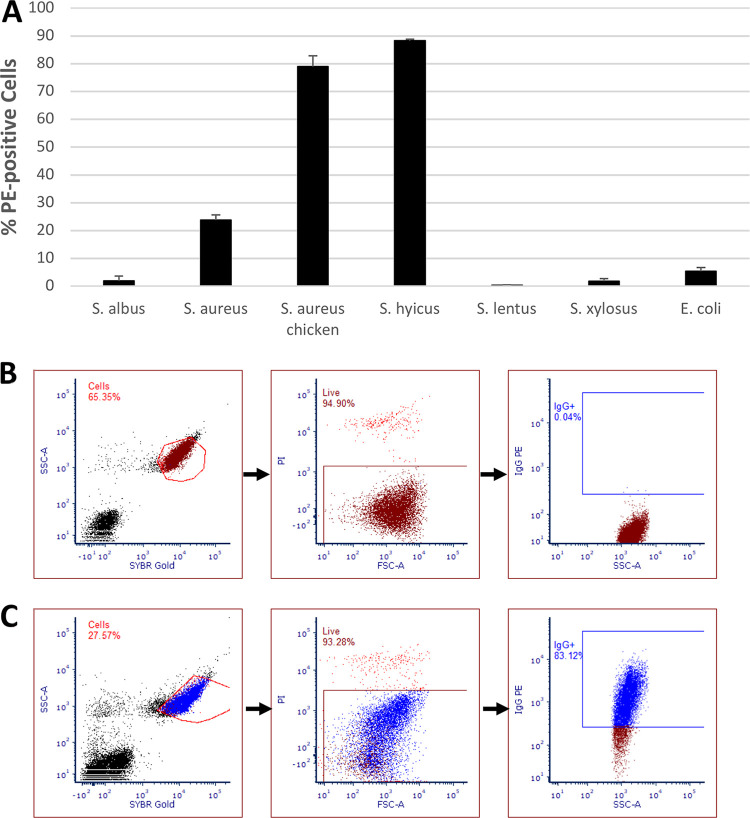
(A) Mean percentages of cells of various *Staphylococcus* strains and E. coli binding to PE-conjugated mouse monoclonal anti-human CD44 antibody compared to unstained control *S. aureus* as measured using FCM (*n* = 2; bars show standard errors of the mean; *P* < 0.01, single-factor ANOVA). (B and C) Gating sequences for flow cytometric analysis of unstained *S. aureus* NCTC 8325 (B) and *S. aureus* chicken ready-meal isolate (C). Cells were distinguished from debris by using a plot of SYBR green fluorescence versus side-scattered light (SSC). Live cells were then distinguished from dead cells using a plot of forward-scattered light (FSC) versus PI fluorescence, and, finally, PE-positive cells were distinguished in a plot of SSC versus PE fluorescence, with the positive gate being drawn based on the fluorescence of unstained *S. aureus* cells.

The mean percentage of live cells for each of the species tested showing a positive signal for phycoerythrin (PE) fluorescence is shown in Fig. 1, along with a typical cytograph showing a negative control (a sample not stained with PE-conjugated mouse monoclonal anti-human CD44 antibody) and a test sample. The PE-positive region was constructed based on the level of PE fluorescence of control samples. Large species-related differences were noted and shown to be significant using a single-factor analysis of variance (ANOVA; *P* < 0.01), with 88.3% of S. hyicus subsp. *hyicus* cells binding the antibody, whereas S. epidermidis (also known as S. albus), S. lentus, and S. xylosus had binding levels below that of the Gram-positive species E. coli (<5%).

### Heat killing affects antibody binding.

**FIG 2:**
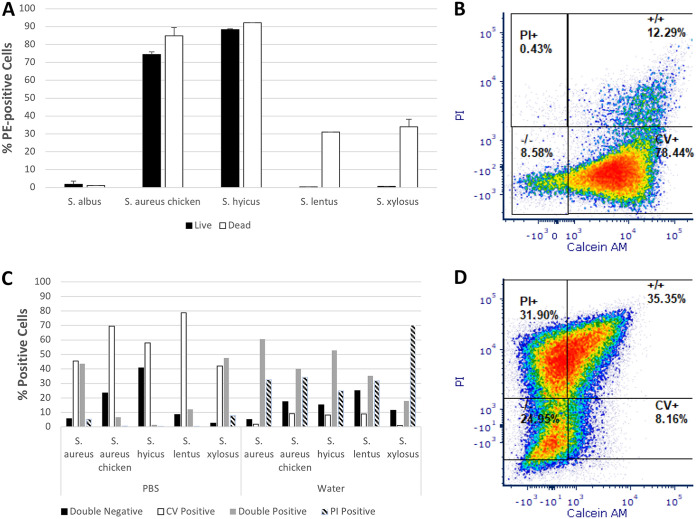
(A) Mean percentages of live or dead cells of various *Staphylococcus* strains displaying binding to PE-conjugated mouse monoclonal anti-human CD44 antibody after exposure to 95°C for 10 min as measured using FCM (*n* = 2; bars show standard errors of the mean; χ^2^ = 50.15061, df = 4, *P* < 0.00001). (C) Percentages of cells stained with PI and calcein violet AM (CV) falling into one of four regions (double negative, calcein violet positive only, double positive, and PI positive only) when analyzed using FCM after storage overnight in water or PBS (*n* = 1; for strains stored in water, χ^2^ = 85.37121, df = 12, *P* < 0.00001; for strains stored in PBS, χ^2^ = 178.30698, df = 12, *P* < 0.00001). (B and D) Flow cytographs of calcein violet versus PI fluorescence of *S. lentus* stored in PBS (B) and water (D).

The mean percentages of PE-positive, propidium iodide (PI)-negative untreated cells versus PI-positive, heat-killed cells are shown in Fig. 2A. A chi-square test found significant differences between observed and expected frequencies (χ^2^ = 50.15061, df = 4, *P* < 0.00001). In S. epidermidis, there is very little difference between the signals of live and dead cells. For the S. aureus chicken ready-meal isolate and S. hyicus subsp. *hyicus*, there was an approximately 5 to 10% increase in the percentage of dead cells showing antibody binding. S. lentus and S. xylosus, whose live cells show little or no binding to the antibody, showed binding of ∼30 to 35% of dead cells.

### Storage of *Staphylococcus* strains in water or PBS affects cell physiology.

For all strains tested, overnight storage in PBS resulted in higher percentages of cells retaining esterase activity compared to controls (Fig. 2B). After storage in water, all strains had increased PI permeability, with greater percentages of double-positive or PI-positive cells. This effect was particularly pronounced for S. lentus, for which ∼79% of cells stored in PBS displayed esterase activity, with only 12.5% PI permeable, whereas for storage in water, esterase-positive cells dropped to ∼9%, with an increase in PI permeability to 67% (Fig. 2C and D). Chi-square tests found significant differences between observed and expected frequencies for both samples stored in water (χ^2^ = 85.37121, df = 12, *P* < 0.00001) and samples stored in PBS (χ^2^ = 178.30698, df = 12, *P* < 0.00001).

### Growth phase influences antibody binding for various strains.

**FIG 3:**
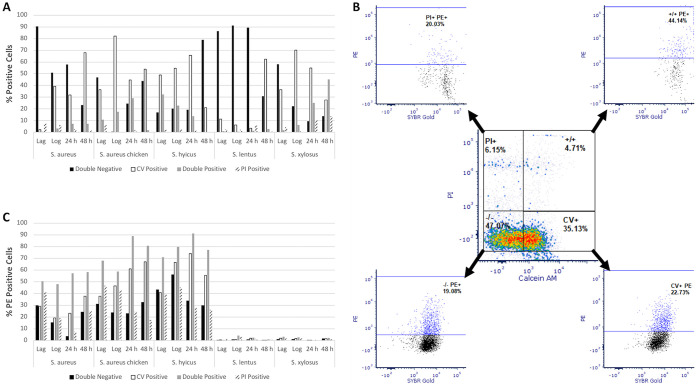
(A) Percentages of cells, as analyzed using FCM of various *Staphylococcus* species, falling into one of four physiological groups based on calcein violet (CV) and PI fluorescence as a function of growth phase (double negative, calcein violet positive only, double positive, and PI positive only; *n* = 1; χ^2^ = 998.60070, df = 57, *P* < 0.00001). (B) Typical flow cytometric analysis scheme to quantify the percentages of cells falling into one of the four physiological groups and measurement of their binding to PE-conjugated mouse monoclonal anti-human CD44 antibody. The data were taken from the analysis of exponential-phase *S. aureus* NCTC 8325. (C) Percentages of cells from each of the physiological groups for each species displaying binding to the anti-human CD44 antibody (*n* = 1; χ^2^ = 116.00109, df = 57, *P* < 0.00001).

Large differences were found for both esterase activity and membrane permeability according to growth phase both within and between strains (Fig. 3A), and a chi-square test showed these to be statistically significant (χ^2^ = 998.60070, df = 57, *P* < 0.00001). For example, in S. aureus NCTC 8325 the greatest percentage of calcein violet-positive cells was seen after 48 h of culture (∼67%), corresponding to the stationary phase, whereas for the S. aureus chicken ready-meal isolate the highest percentages of calcein violet-positive cells were found during the exponential growth phase (∼80%). The majority of S. lentus cells were neither esterase positive nor PI permeable during lag and log phases of culture. However, at 48 h ∼60% of the cells displayed esterase activity. *S. xylosus* was the only strain for which >50% of cells showed membrane permeability (double positive and PI positive) after 48 h of culture.

Regardless of growth phase and physiological status, less than 3% of S. lentus and S. xylosus cells displayed PE fluorescence from binding of the anti-human anti-CD44 antibody (Fig. 3C). In the other three strains, high levels of antibody binding were recorded, and the percentages of PE-positive cells varied according to strain, cell culture phase, and cell physiological status. As a general trend for these three strains, double-positive cells gave the highest levels of antibody binding, generally followed by calcein violet-positive cells. The highest percentages of PE-positive cells were noted for *S. hyicus* subsp. *hyicus* and S. aureus chicken ready-meal isolates after 24 h of growth. In some cases, the difference between the percentages of PE-positive cells from physiological groups from each phase was dramatic: in the S. aureus chicken ready-meal isolate at 24 h, ∼90% of the double-positive cells showed antibody binding compared to ∼20% of the double-negative cells. A chi-square test revealed significant differences in the levels of antibody binding of the physiological groups of the strains (χ^2^ = 116.00109, df = 57, *P* < 0.00001).

### Cell stressor treatments affect antibody binding of *S. aureus*.

**FIG 4:**
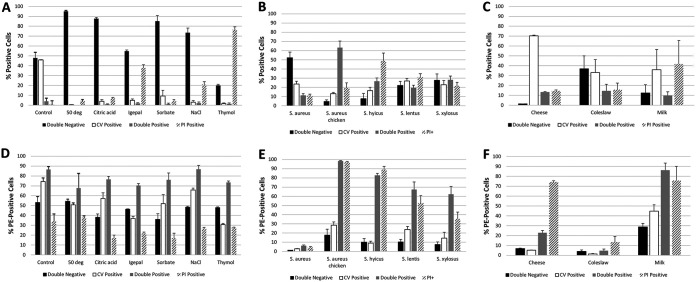
(A) Mean percentages of cells of *S. aureus* chicken ready-meal isolate falling into one of four physiological groups (double negative, calcein violet positive only, double positive, and PI positive only) based on calcein violet (CV) and PI fluorescence after treatment with 0.09% (wt/vol) potassium sorbate, 12% NaCl, 0.09% sodium citrate, 0.5 % IGEPAL CO-630, or 0.25% (wt/vol) thymol or after heat treatment at 50°C for 30 min and analyzed using FCM (*n* = 3; χ^2^ = 436.45846, df = 18, *P* < 0.00001), as well as the binding of anti-human CD44 antibody by cells from these groups (D; *n* = 3; χ^2^ = 29.53591, df = 18, *P* = 0.04221). (B) Percentages of cells as analyzed using FCM of various *Staphylococcus* species falling into one of four physiological groups after incubation on stainless-steel surfaces for 24 h at 37°C (*n* = 5; χ^2^ = 154.15338, df = 12, *P* < 0.00001) and binding of anti-human CD44 antibody by cells from these groups (E; *n* = 5; χ^2^ = 21.05560, df = 12, *P* = 0.04957). (C) Percentages of cells as analyzed using FCM of *S. aureus* chicken ready-meal isolate spiked into cheese (*n* = 2), coleslaw (*n* = 3), or milk (*n* = 3) falling into one of four physiological groups based on calcein violet (CV) and PI fluorescence (χ^2^ = 77.37324, df = 6, *P* < 0.00001), as well as the binding of anti-human CD44 antibody by cells from these groups (F; χ^2^ = 44.72049, df = 6, *P* < 0.00001). In all cases, error bars indicate the standard errors of the mean.

The physiological profiles of S. aureus chicken ready-meal isolate cells differed between treatments (Fig. 4A), and these were found to be statistically significant using a chi-square test (χ^2^ = 436.45846, df = 18, *P* < 0.00001). All stressor treatments resulted in reductions in esterase-positive cells compared to the control. An almost total reduction in esterase-positive cells was noted for the following conditions: heating to 50°C for 30 min, treatment with 0.09% sodium citrate, and treatment with 0.09% (wt/vol) potassium sorbate and 12% NaCl. Large increases in permeability to PI were found after exposure to 0.5% IGEPAL CO-630, 12% NaCl, and 0.25% (wt/vol) thymol. Plate count data indicated a reduction of between 1 and 3 logs for all treatments, with exposure to thymol leading to the largest reduction (data not shown). For both the control and experimental groups, the PI-positive subpopulation showed the lowest percentage of antibody-binding cells, with between 15 and 40% of cells positive for PE signal (Fig. 4B). Esterase-positive (either single positive or positive for both calcein violet and PI) subpopulations generally displayed the highest levels of antibody binding. Differences in antibody binding among variously treatments were found to be statistically significant using a chi-square test (χ^2^ = 29.53591, df = 18, *P* = 0.04221).

### Antibody binding of *Staphylococcus* species is affected by incubation on stainless-steel surfaces.

The physiological profiles of *Staphylococcus* spp. spread onto stainless-steel surfaces differed between strains (Fig. 4B), and these differences were found to be statistically significant using a chi-square test (χ^2^ = 154.15338, df = 12, *P* < 0.00001). There were also differences between the profiles for each strain established during growth phase experiments (compare Fig. 4B and Fig. 3A). The majority of S. aureus NCTC 8352 cells were PI and calcein violet negative, and the majority of S. aureus chicken ready-meal isolate cells were PI and calcein violet positive, whereas the reductions in cell viability as measured by plate counting were similar (6 log units) (data not shown). There was an approximately equal division of cells into the four subpopulations for S. lentus and S. xylosus. The former was the most resistant strain to the stresses of transfer and incubation on a stainless-steel surface, with only a 1-log reduction in viability, whereas the latter showed a 3-log reduction. *S. hyicus* subsp. *hyicus* had the highest percentage of PI-positive cells (∼50%).

Transfer to stainless-steel surfaces almost completely eliminated antibody binding by S. aureus NCTC 8325, with <5% of the subpopulations binding to anti-human CD44 antibody (Fig. 4E). For the other strains, it was predominantly the two permeabilized populations that bound the antibody. Surprisingly, surface-adhering S. lentus and *S. xylosus* reacted with the antibody, while neither of these species bound antibody during growth in liquid culture (compare Fig. 4E and 3C). Differences in antibody binding among the strains were found to be statistically significant using a chi-square test (χ^2^ = 21.05560, df = 12, *P* = 0.04957).

### Antibody binding by *S. aureus* differs in various food systems.

Combined data for antibody binding for S. aureus in coleslaw, milk, and grated cheese samples are presented in Fig. 4F. For milk and grated cheese, plate count data indicated that the majority of the bacteria present were the S. aureus chicken ready-meal isolate, while in the case of coleslaw ∼50% of those present were the native microflora. After overnight incubation at refrigeration temperature, ∼70% of cells present in cheese were esterase positive, whereas in coleslaw and milk this figure was ca. 30 to 40% (Fig. 4C). The percentage of PI-permeable cells was highest in milk (∼50%). These differences were statistically significant when analyzed using a chi-square test (χ^2^ = 77.37324, df = 6, *P* < 0.00001). In cheese there was a dramatic difference between the percentages of cells from each of the physiological groups showing antibody binding, with 70% of PI-positive cells reacting with the antibody compared to 5 to 20% of cells from the other groups (Fig. 4F). In milk this difference was less pronounced, but it was still the two permeabilized groups that showed greater antibody binding. Since the S. aureus chicken ready-meal isolate comprised only 50% of the microflora in the coleslaw, it is not surprising that the percentages of cells binding antibody were much reduced, but even in this foodstuff it was the permeabilized cells that bound in greater numbers to the antibody. Differences in antibody binding among the cells inoculated into the three foodstuffs were found to be statistically significant using a chi-square test (χ^2^ = 44.72049, df = 6, *P* < 0.00001).

### Polyclonal antibodies raised against other bacteria bind to *Staphylococcus* species.

**FIG 5:**
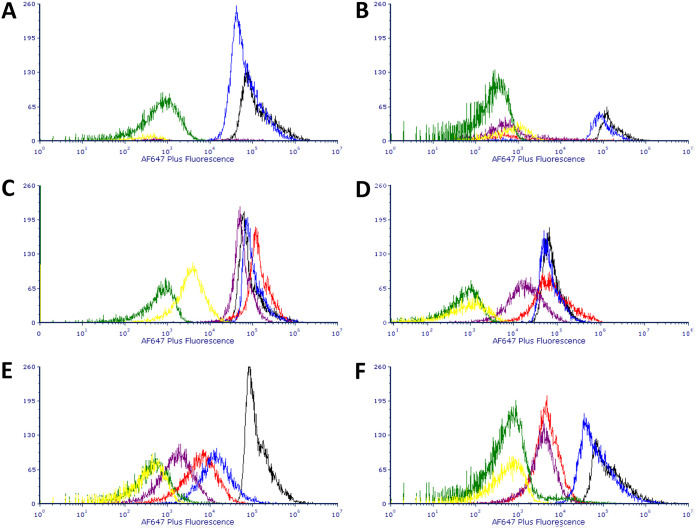
(A to F) Overlays of histograms for the Alexa Fluor 647 Plus fluorescence of S. epidermis (A), *S. capitis* (B), *S. aureus* NCTC 8325 (C), *S. hyicus* (D), *S. aureus* pepper isolate (E), and *S. xylosus* (F) stained with one of two primary anti-*Enterococcus* commercial antibodies combined with Alexa Fluor 647 Plus-conjugated anti-rabbit secondary antibody or Alexa Fluor 647 Plus-conjugated anti-rabbit secondary antibody alone. Color codes for each of the histograms: black, E. faecium stained with primary Abcam and secondary antibodies; red, test species stained with Abcam primary and secondary antibodies; blue, E. faecium stained with 2B Scientific primary and secondary antibodies; purple, test species stained with 2B Scientific primary and secondary antibodies; green, E. faecium stained with secondary antibody only; yellow, test species stained with secondary antibody only.

Figure 5 shows the binding in six individual panels of S. epidermidis, S. aureus NCTC 8325, S. aureus mixed pepper isolate, S. capitis subsp. *capitis*, S. hyicus subsp. *hyicus*, and S. xylosus to the pair of primary anti-*Enterococcus* commercial antibodies combined with Alexa Fluor 647 Plus-conjugated anti-rabbit secondary antibody and to Alexa Fluor 647 Plus-conjugated anti-rabbit secondary antibody alone. *Staphylococcus* strains that displayed Alexa Fluor 647 Plus fluorescence similar to (and occasionally higher than) that for the target species included S. epidermidis and S. aureus NCTC 8325 when both primary and secondary antibodies were present (reactions were similar for both commercial antibodies) and S. hyicus subsp. *hyicus* when the Abcam antibodies was present. Strains which displayed Alexa Fluor 647 Plus fluorescence greater than that of the secondary-antibody-only control but less than that of the target species primary and secondary antibodies included the S. aureus mixed pepper isolate, *S. xylosus* ATCC 700404 (for both commercial antibodies), and *S. hyicus* subsp. *hyicus* for the 2B Scientific antibody. The reaction of *S. capitis* subsp. *capitis* when primary antibody was included along with secondary antibody was almost identical to that when secondary antibody alone was present, and, in spite of the presence of “tails,” peaks were separated from those of target species by >2-log decades. It was only in *S. capitis* subsp. *capitis*, as well as in S. aureus NCTC 8325, that Alexa Fluor 647 Plus fluorescence was markedly greater for secondary antibody alone than for the target species in the presence of secondary antibody alone (see yellow histogram in Fig. 5B and C). With the exception of S. epidermidis, when both primary and secondary antibodies were present, fluorescence distributions were unimodal.

**FIG 6:**
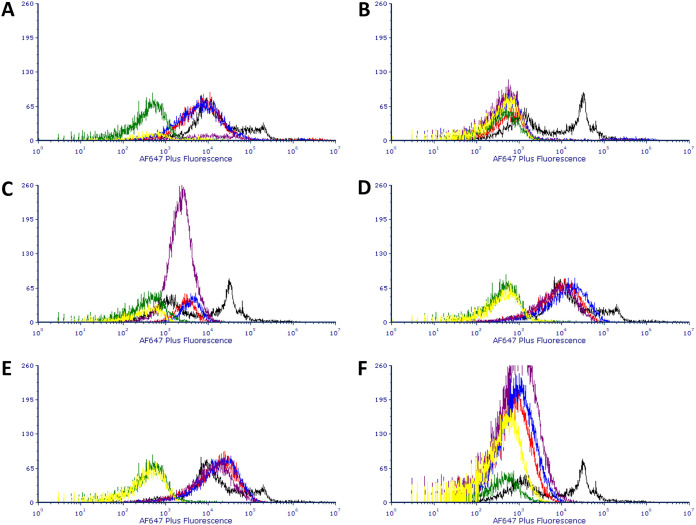
(A to F) Overlays of histograms for the Alexa Fluor 647 Plus fluorescence of S. epidermis (A), *S. capitis* (B), *S. aureus* NCTC 8325 (C), *S. hyicus* (D), *S. aureus* pepper isolate (E), and *S. xylosus* (F) stained with one of three primary anti-*Bacillus* endospore commercial antibodies combined with Alexa Fluor 647 Plus-conjugated anti-rabbit secondary antibody or Alexa Fluor 647 Plus-conjugated anti-rabbit secondary antibody alone. Color codes for each of the histograms: black, B. cereus ensdospores stained with Genway primary and secondary antibodies; red, test species stained with Genway primary and secondary antibodies; blue, test species stained with EastCoast Bio primary and secondary antibodies; purple, test species stained with ViroStat primary and secondary antibodies; green, *B. cereus* stained with secondary antibody only; yellow, test species stained with secondary antibody only.

Binding by three commercial anti-*Bacillus* antibodies to *Staphylococcus* species is presented in Fig. 6. Cultures of a positive control of Bacillus cereus NCTC 7464 spores displayed a bimodal pattern of binding to all three commercial antibodies, presumed to reflect the presence of spores in the preparation with different levels of epitope expression. *S. capitis* subsp. *capitis* had low reactivity to the primary antibodies. S. aureus NCTC 8325 and *S. xylosus* had reactivity to the primary antibodies, with a peak of fluorescence intensity similar to the lower of the target species’ peaks. The remaining species had fluorescence peaks that overlapped the higher peak of the target species. In general, the levels of binding of each of the commercial antibodies to a given *Staphylococcus* species were very similar, which may indicate that these preparations of polyclonal antibody are similar in terms of isotype (compare the correspondence of the red, blue, and purple histograms in each of the panels in Fig. 6). In this series of experiments, only S. epidermidis displayed (slightly) higher binding to the secondary antibody alone.

### Purified mouse IgG binds to *S. aureus*.

**FIG 7:**
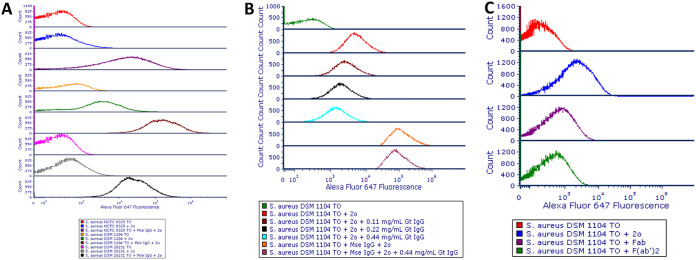
(A to C) Overlays of histograms for the Alexa Fluor 647 fluorescence of *S. aureus* NCTC 8325, DSM 1104 and DSM 20231 incubated with mouse IgG, followed by staining with Alexa Fluor 647-conjugated goat anti-mouse IgG secondary antibody (A); *S. aureus* DSM 1104 incubated with various concentrations of purified goat IgG, followed by staining with mouse IgG and/or goat anti-mouse Alexa Fluor 647-conjugated secondary antibody (B); and *S. aureus* DSM 1104 cells incubated with whole, Fab, or F(ab′)_2_ fragment goat anti-mouse AF 647-conjugated antibodies (C). 2o, secondary antibody; Fab, Fab fragment goat anti-mouse IgG antibody; F(ab′)2, F(ab′)_2_ fragment goat anti-mouse antibody; Gt IgG, purified goat IgG; Mse IgG, mouse IgG; ITO, thiazole orange.

All three S. aureus strains bound purified mouse IgG and showed an increase in Alexa Fluor 647 fluorescence intensity compared to the negative control. S. aureus DSM 1104 also bound goat anti-mouse fluorescence-labeled IgG antibody alone and to a greater degree than did the other S. aureus strains (Fig. 7A).

### Blocking of binding of purified mouse IgG to *S. aureus* with goat IgG.

S. aureus DSM 1104 incubated with increasing concentrations of purified goat IgG as a blocking mechanism for binding of mouse IgG showed only a slight decrease in fluorescence when subsequently incubated with goat anti-mouse Alexa Fluor 647-conjugated secondary antibody compared to controls (Fig. 7B). Even the highest concentrations of goat IgG used had little effect on the fluorescence intensity of cells incubated with mouse IgG followed by goat anti-mouse AF647-labeled antibody. Prior incubation of S. aureus DSM 1104 with goat anti-mouse IgG, followed by staining with secondary antibody, only slightly reduced the binding of secondary antibody to cells, which showed fluorescence intensities of ∼1 log above that of unstained control cells. Incubation of S. aureus DSM 1104 cells with Fab or F(ab′)_2_ fragment goat anti-mouse AF 647-conjugated antibodies resulted in reduced fluorescence compared to the (whole) goat anti-mouse secondary antibody, suggesting that the binding of the secondary antibody to cells was through Fc-SpA interactions (Fig. 7C).

### Blocking *S. aureus* binding of polyclonal antibodies raised against other bacterial species.

**FIG 8:**
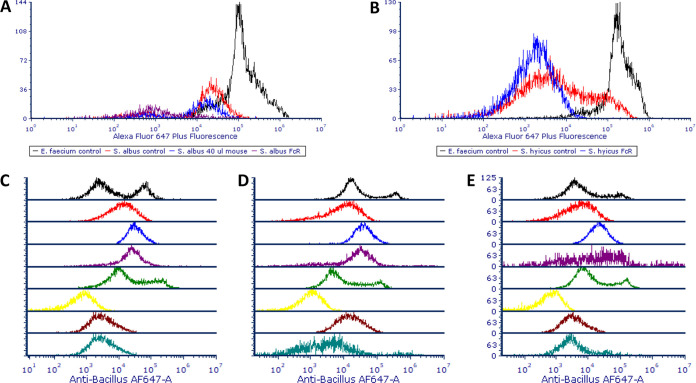
(A) Overlays of the Alexa Fluor 647 Plus fluorescence of E. faecium and S. epidermidis stained with rabbit anti-*Enterococcus* primary antibody, followed by goat anti-rabbit Alexa Fluor 647 Plus and S. epidermidis preincubated with 40 μl per 100 or FcR blocking reagent prior to staining. (B) *E. faecium* and *S. hyicus* stained with primary and secondary antibodies or preincubated with FcR blocking reagent prior to staining. (C to E) Overlays of histograms for the Alexa Fluor 647 fluorescence of various strains stained with Genway (C), EastCoast Bio (D), or ViroStat (E) primary anti-*Bacillus* endospore commercial antibodies combined with Alexa Fluor 647 Plus-conjugated anti-rabbit secondary antibody or Alexa Fluor 647 Plus-conjugated anti-rabbit secondary antibody alone. Color codes for panels C to E: black, *B. cereus*; red, *S. hyicus*; blue, *S. aureus*; purple, S. epidermidis; green, *B. cereus* preincubated with FcR blocking reagent; yellow, *S. hyicus* preincubated with FcR blocking reagent; brown, *S. aureus* preincubated with FcR blocking reagent; aquamarine, S. epidermidis preincubated with FcR blocking reagent.

Figure 8 shows the blocking of SpA-mediated binding of polyclonal antibodies raised against non-*Staphylococcus* species. In the case of the Abcam anti-*Enterococcus* antibody, the use of a 2:5 dilution of the FcR product in SB eliminated the majority of the overlap in Alexa Fluor 647 Plus fluorescence profiles of S. epidermidis and S. *hyicus* subsp. *hyicus* with Enterococcus faecium ATCC 19434. In the case of the Genway anti-*Bacillus* antibody, FcR blocker reduced the binding of primary antibody to *S. hyicus* subsp. *hyicus* below the level of that of the B. cereus NCTC 7434 positive control’s lower fluorescence peak, while the signals for S. aureus NCTC 8325 and S. epidermidis, although still being reduced by ∼1-log unit, overlapped slightly with the lower tail of B. cereus’ peak. This pattern was also repeated for the other commercial anti-*Bacillus* antibodies, except for the EastCoast Bio antibody and its interaction with S. aureus NCTC 8325. In this case, S. aureus’ signal fell between that of B. cereus’ pair of peaks.

## DISCUSSION

Any antibody-based detection assay for bacteria must be demonstrably specific, with minimal cross-reactivity with nontarget species (16). However, a significant source of cross-reactivity is SpA-mediated binding of antibodies to *Staphylococcus* species. In this study, binding by SpA-expressing strains of a range of commercial antibodies to non-*Staphylococcus* targets (a phenomenon behind much of the nonspecificity of antibody-mediated detection assays) was characterized using FCM, a technique that allows the measurement of the fluorescence emitted by large numbers of individual cells. Through the use of FCM and the recording of fluorescence data from individual cells, we have demonstrated a high degree of heterogeneity in SpA-mediated binding of antibodies: not every S. aureus or S. hyicus subsp. *hyicus* cell in a suspension shows the same degree of binding to antibody. The degree of binding is strain, physiological state, growth phase, and matrix dependent.

At the outset of the present study, the binding of a randomly chosen mouse monoclonal anti-human CD44 antibody (isotype IgG2) to a pair of S. aureus strains and four *Staphylococcus* species was measured using FCM. This binding was likely due to the presence of SpA. Although S. epidermidis, S. lentus ATCC 738152, and *S. xylosus* ATCC 700404 did not bind the antibody (and are not associated with expression of SpA) (17), S. aureus NCTC 8325, an S. aureus chicken ready-meal isolate, and *S. hyicus* subsp. *hyicus* DSM 20459 displayed proportions of cells binding to the antibody above the levels of unstained control to 25, 80, and 85% of live cells, respectively. It was not surprising to encounter differences in the binding of two S. aureus strains to antibody: strain-related differences have been reported in the past (6, 13, 18), and some authors have not encountered cross-reactivity associated with certain S. aureus strains during the development of antibody-based detection assays (11). The fact that <100% of cells bind the antibody may be explained by the growth-phase-related expression of SpA, as the protein is actively synthesized during exponential growth (6). Early-exponential-phase cultures derived from saturated overnight cultures were used in the present report. Since *S. hyicus* subsp. *hyicus* appeared to strongly bind antibody and since a previous study found that 93.6% of *S. hyicus* species expressed SpA (19), we recommend that testing for cross-reactivity with *S. hyicus* be included in the development of any new antibody-based assay against non-*Staphylococcus* targets, especially in the context of clinical samples.

A number of simulated food-processing treatments were applied to exponential-phase cultures of the S. aureus chicken ready-meal isolate. A previous study by Kennedy et al. (15) found that certain simulated food-processing treatments produced changes in the intensity of binding of a pair of commercial mouse IgM anti-S. aureus antibodies to an S. aureus food isolate and S. aureus ATCC 8325. These alterations depended on the nature of the treatment; the organic acids citric acid and oxalic acid resulted in a significant decrease in antibody binding, possibly through damage to the cell membrane and intracellular enzymes, whereas heat treatments to simulate mild to more severe heat processing treatments (from 50 to 80°C) resulted in only small differences in the degree of antibody binding. Alterations caused by treatments used here (0.09% [wt/vol] potassium sorbate; 12% NaCl, 0.09% sodium citrate, 0.5% IGEPAL CO-630, 0.25% [wt/vol] thymol, and heat treatment at 50°C for 30 min) to cell membrane integrity and intracellular esterase activity were measured using FCM, and the percentages of cells falling into the four physiological categories described above were recorded (Fig. 4). As for the data from the growth phase and cell storage experiments, cells displaying esterase activity, regardless of treatment, tended to show the highest likelihood of SpA-mediated antibody binding. Permeabilized cells (PI-only positive) showed the least likelihood of binding antibody. It would appear, therefore, that an intact membrane and cellular vitality, as reflected by intracellular esterase activity, are important for the maintenance of SpA-mediated antibody binding. The variability in S. aureus SpA-mediated antibody binding as a cause of false-positive signals in antibody-mediated detection assays may be a function of this phenomenon; in food systems, for example, cells exist in a variety of physiological states owing to the preservatives found in food or the processing that the foodstuff or its ingredients have experienced. An extension of this is the idea that in the context of enrichment, where a dilution from a sample may be added to a broth designed to favor the growth of the organism being detected but where S. aureus may recover its vitality (or indeed multiply), S. aureus SpA-mediated antibody binding could pose an increased risk of giving rise to false-positive signals. Other researchers have reported such problems with enrichment media in the context of the detection of bacteria using antibodies (1).

The most unexpected result arising from the experiment in which 1-ml aliquots of exponential-phase cultures were applied to stainless-steel surfaces, allowed to air dry, and left for 24 h before having their physiology and antibody binding measured by FCM was the increase in the binding of the non-SpA-expressing strains S. lentus and *S. xylosus* to antibody. This was in the context of S. aureus NCTC 8325 losing its ability to bind antibody. It could be that the manner of S. lentus and *S. xylosus* binding to antibody was similar to the damage-mediated, nonspecific binding found in a previous study by this group, where cell damage was shown to increase antibody signal above that of healthy control cells (15). S. lentus and *S. xylosus* also displayed the smallest log reductions after incubation on stainless steel compared to the other strains, as measured by plate counting, and FCM data showed that ∼50% of the cells maintained esterase activity. It could be speculated that the mild damage suffered by cells of these strains was responsible for nonspecific binding of antibody, whereas the 6-log reduction and cytometric profile seen in the case of S. aureus NCTC 8325 indicated so severe a level of damage to the structure of cells that SpA-mediated antibody binding was largely eliminated. For the S. aureus chicken ready-meal isolate and *S. hyicus* subsp. *hyicus*, it was the two PI-positive populations that bound antibody. This is in contrast to data from the growth phase and simulated food processing treatment experiments, wherein cells displaying esterase activity showed the highest likelihood of SpA-mediated antibody binding. It would seem that the damage induced in cells during drying and adherence to stainless-steel surfaces exerts a different effect on SpA expression and maintenance than occurs during normal growth in broth, storage, or food processing. This experiment illustrates the complexity of both SpA-mediated antibody binding (in the case of S. aureus and *S. hyicus* subsp. *hyicus*) and nonspecific damage-induced binding (in the case of S. lentus and S. *xylosus*) in “real world” scenarios such as cell adhesion to dry stainless-steel surfaces. The development of antibody-mediated detection methods for bacteria must take this complexity into account to ensure that false-positive and false-negative results are minimized (20).

Another “real world” scenario was examined: the expression of SpA by cells of the S. aureus chicken ready-meal isolate spiked into coleslaw, milk, and grated cheese and left overnight at refrigeration temperature. These cells reacted differently to being spiked into the different foods in terms of their percentages falling into each of the four physiological groups (Fig. 4). For example, ∼70% of the cells present in cheese were esterase positive, while ∼50% of the cells in milk were permeabilized. Expression of SpA in each of the food systems varied according to cell physiological status. In general, and similar to the data yielded by the surface spiking experiment, permeabilized cells showed greater antibody binding. This points toward a conclusion reached in the Kennedy et al. (15) study, where it was recommended that a cell membrane permeability dye such as PI be used to remove membrane-damaged cells from the final count in FCM antibody-mediated detection assays in order to reduce the problem of false positives.

In the latter part of this study, the binding of *Staphylococcus* strains to commercial antibodies raised against other bacterial species (*Enterococcus* sp. and *Bacillus* sp. spores), as well as the fluorescence-labeled secondary antibody used to mark primary antibody-tagged cells, was assessed, and the findings suggest that rigorous testing of commercial antibodies against *Staphylococcus* spp. and other nontarget species must be carried out during the development of assays, something that is not always reported to have been carried out (see, for example, references 21 and 22). All commercial primary antibodies tested in this present study were polyclonal and raised in rabbits. One of the two anti-*Enterococcus* antibodies was stated by its manufacturers to be of isotype IgG (Abcam). No information on isotype was provided by the manufacturers of the other anti-*Enterococcus* antibody or the three anti-*Bacillus* spore antibodies. The secondary antibody was polyclonal goat IgG anti-rabbit Alexa Fluor 647 Plus conjugated. What emerged from these experiments is a complex picture (Fig. 5), with not all SpA-expressing species showing strong antibody binding to mouse IgG and one species not known to express SpA showing strong binding (S. epidermidis). For S. aureus NCTC 8325 and S. epidermidis, the level of binding to primary and secondary antibodies in combined staining was above that of the positive control of target species in the case of both commercial anti-*Enterococcus* antibodies, and for *S. hyicus* subsp. *hyicus* this was the case for the Abcam antibody. For this same combination and for both brands of antibody, other strains showed antibody binding above that of the secondary-antibody-only-stained target species negative control but below that of the target species-S. aureus mixed pepper isolate and *S. xylosus. S. capitis* subsp. *capitis* and S. aureus NCTC 8325 gave signals for secondary antibody alone greater than that of the target species. It is interesting to note that fluorescence distributions of *Staphylococcus* strains binding the combination of primary anti-*Enterococcus* and secondary antibodies were unimodal, with the exception of S. epidermidis, and that in experiments with anti-*Bacillus* antibodies this strain was the only one that showed greater binding to secondary antibody alone than did the control secondary-antibody-only-stained target species. These data could be a reflection of the fact that this strain’s binding of antibody was by some mechanism other than through SpA. It was two SpA-expressing strains that showed binding to the trio of commercial anti-*Bacillus* spore antibodies equal to that of the higher peak of the target species: S. aureus mixed pepper isolate and *S. hyicus* subsp. *hyicus*. The other SpA-expressing strain, S. aureus NCTC 8325, yielded a signal intensity similar to that of the lower peak of the target species, as did *S. xylosus*. On the other hand, in their binding to purified mouse IgG, the three S. aureus strains tested—S. aureus NCTC 8325, DSM 1104, and DSM 20231—behaved predictably, showing an increase in fluorescence signal compared to the negative control. Only S. aureus DSM 1104 bound goat IgG antibody, demonstrating that SpA-expressing strains’ reactivity with IgG antibodies of this species could be strain dependent. These experiments with commercial antibodies and purified serum demonstrate the complexity of predicting which particular strains will yield unwanted positive signal in the context of a given antibody-based assay. It is recommended that in the development of any new antibody-based microbial detection assay a variety of SpA-expressing strains are tested for their binding of both primary and secondary antibodies. A non-SpA-expressing *Staphylococcus* species, such as S. epidermidis, should also be included to investigate the possibility of non-SpA-mediated nontarget antibody binding.

The final part of this study involved attempts to block the unwanted SpA-mediated binding by *Staphylococcus* species of antibodies targeting non-*Staphylococcus* species using three products: normal rabbit serum, mouse IgG isotype control, and FcR blocking reagent. Attempts were also made to block the nonspecific binding of S. aureus NCTC 8325, DSM 1104, and DSM 20231 strains to mouse IgG by incubation of the cells with goat IgG. Of the first three products, only FcR blocking reagent reduced or eliminated the binding of mouse IgG antibody raised against *Enterococcus* to S. aureus NCTC 8325, S. epidermidis, and S. *hyicus* subsp. *hyicus*. In the case of anti-*Bacillus* IgG antibody raised in mice, the binding of *S. hyicus* subsp. *hyicus* was reduced below the levels of the positive control, and the intensity of antibody signal from the binding of S. aureus NCTC 8325 and S. epidermidis was reduced by ∼1-log unit, exhibiting a slight overlap with the lower of the two positive controls’ peaks. For one of the three antibody brands (EastCoast Bio), while the SpA-mediated binding of antibody by S. aureus NCTC 8325 was reduced by preincubation with FcR blocking reagent, the signal fell between that of the B. cereus positive control’s pair of peaks. This level of brand- and species-dependent variability in the blocking produced by the FcR reagent suggests that extensive validation of the product’s utility for each antibody detection method is needed. FcR blocking reagent is described by the manufacturers as a product for the blocking of unwanted binding of antibodies to human Fc receptor-expressing cells and targets the epitopes on Fc receptors that bind the Fc regions of antibodies. It was thought that this product could also block the regions on SpA responsible for Fc binding and, indeed, the product seems to have achieved this effect. A similar approach by other authors proved effective in blocking the binding of SpA to von Willebrand factor, where the binding of fluorescein isothiocyanate-labeled von Willebrand factor to S. aureus was inhibited by anti-SpA antibodies (23).

The use of normal rabbit serum as a blocking reagent gave rise to a total absence of signal from the species-specific polyclonal antibody, presumably through the blocking of all epitopes on the bacteria recognized by the species-specific antibodies, and produced an unreliable blocking of S. aureus binding to primary and secondary antibodies. The use of mouse serum only weakly diminished S. aureus antibody binding, possibly through insufficient “covering” of SpA. Incubation with goat serum IgG antibody as a blocker prior to incubation with mouse IgG produced only a minimal decrease in fluorescence when cells were subsequently stained with goat anti-mouse Alexa Fluor 647-conjugated antibody. Furthermore, goat serum IgG failed to reduce the binding of goat IgG secondary antibody alone to S. aureus DSM 1104. In contrast, the elimination of false-positive signals in the case of anti-*Enterococcus* and anti-*Bacillus* assays produced by FcR blocking reagent showed that this product has real utility in eliminating SpA-mediated binding in certain cases.

While some researchers have devised methods to overcome the issue of *Staphylococcus* binding to their species-specific antibody (e.g., fluorescent wheat germ agglutinin was included as a counterstain to differentiate false-positive antibody-binding S. aureus from the target species [4]), such solutions are assay specific. A more universal approach, which would bypass issues with the SpA-mediated binding of antibodies, would be to use either antibody fragments lacking the Fc region [Fab′, Fv, F(ab)_2_, or Fab], recombinant antibodies, or nonimmunoglobulins such as aptamers (24, 25). Unfortunately, the majority of commercial antibodies against bacterial species are not available in Fab′, Fv, F(ab)_2_, or Fab forms, and the development of recombinant antibodies and aptamers is expensive and complex. As such, we recommend as a practical and widely applicable solution the use of FcR blocking reagent in the development of any antibody-based detection method where SpA-expressing *Staphylococcus* species are likely to present a problem of false positives.

## MATERIALS AND METHODS

### Bacterial strains.

The following strains were used in this study: Bacillus cereus NCTC 7464 purified endospores as per other workers (26); Enterococcus faecalis ATCC 19433; E. faecium ATCC 19434; Escherichia coli ATCC 1175; S. epidermidis; S. aureus NCTC 8325; S. aureus DSM 1104; S. aureus DSM 20231; two S. aureus food isolates, one originating from mixed pepper and the other from a chicken ready-meal; *S. capitis* subsp. *capitis* ATCC 35661; *S. hyicus* subsp. *hyicus* DSM 20459; S. lentus ATCC 738152; and *S. xylosus* ATCC 700404. All stocks were preserved in 1 ml of Cryoinstant cryotubes (VWR, Leuven, Belgium) at –20°C, with colonies for working stocks taken from nutrient agar (NA; Thermo Fisher Scientific, Dublin, Ireland) slants and grown on NA in petri dishes (or lysogeny broth [LB] agar in the case of E. coli; Thermo Fisher Scientific). All liquid suspensions of the organisms were grown in nutrient broth (NB; Thermo Fisher Scientific; LB in the case of E. coli), with *Enterococcus* spp. being grown at 30°C and *Staphylococcus* spp. and E. coli being grown at 37°C. For overnight cultures, one colony of the species in question was placed in 5 ml of NB (or LB in the case of E. coli) in a 25-ml flask and shaken at 240 rpm. Unless otherwise stated, 1/100 dilutions in NB of overnight suspensions in a final volume of 20 ml in 250-ml flasks harvested after 2 h of growth at the relevant temperature with shaking at 240 rpm (corresponding to early exponential phase) were used as experimental material.

### Bacterial growth curves.

Overnight suspensions of the above species were diluted to 1:50 or 1:25 in NB (or LB in the case of E. coli) and transferred to individual wells of sterile, clear, flat-bottomed polystyrene 96-well plates (Sarstedt, Drinagh, Ireland), with a final volume of 200 μl. The optical density at 600 nm (OD_600_) of these suspensions was measured using a Synergy HT microplate reader (BioTek Instruments, Inc., Winooski, VT). Microplates preheated to the desired temperature were incubated at 37°C (or 30°C in the case of *Enterococcus* spp.) over the course of 48 h, with readings taken every 30 min after shaking at intensity 3 for 5 s (cultures were not continuously shaken). Absorbance readings were taken with a top probe vertical offset of 1 mm. Data were exported as CSV files, and growth curves were constructed using a spreadsheet package (see below). Lag, exponential, stationary, and death phases were identified for each strain based on time versus OD_600_ growth curves, with aliquots for growth-phase-related experiments removed from cultures at various time points based on these curves.

Enumeration of points along growth curves were carried out using plate counting, with serial dilutions performed in peptone water (Thermo Fisher Scientific). Then, 100-μl aliquots were spread onto surfaces of NA (or LB in the case of E. coli) petri dishes, and incubation was carried out at the relevant temperature for each organism. Counts between 30 and 300 CFU per plate were taken after overnight incubation.

### Antibodies, fluorescent dyes, and staining regimes for flow cytometry.

PE-conjugated mouse monoclonal anti-human CD44 antibody (isotype IgG2a; catalog number ab82529; Abcam, Cambridge, UK) was used as a model to test for SpA-mediated binding of *Staphylococcus* strains to specific IgG antibodies. Titrations with this antibody against S. aureus NCTC 8325 indicated that a dilution of 1:50 gave the highest percentage of PE-positive cells (data not shown). To demonstrate SpA-mediated binding to polyclonal antibodies raised against microbial species, a pair of unconjugated antibodies against E. faecium were used (anti-*Enterococcus* antibody, catalog no. ab68540 [Abcam]; *Enterococcus* species, polyclonal antibody, catalog no. MBS315075 [2B Scientific, Oxford, UK]). Both of these antibodies were raised in rabbits. The Abcam anti-*Enterococcus* antibody is described by its manufacturers as belonging to isotype IgG. After a series of titrations against E. faecalis ATCC 19433 and E. faecium ATCC 19434, both antibodies were used at a 1:50 dilution. Three antibodies against *Bacillus* spores were also tested for their reactivity with *Staphylococcus* species: Omnitope anti-*Bacillus* spores antibody, catalog no. 3401 (ViroStat, Portland, ME); anti-*Bacillus* antibody, catalog number JP003 (EastCoast Bio, North Berwick, ME); and anti-*Bacillus* spores antibody, catalog no. GWB-W872F (Genway Biotech, Inc., San Diego, CA). All three antibodies are polyclonal, raised in rabbits, and described as having as immunogen spores of B. cereus and B. subtilis. These antibodies were used at dilutions of 1:50.

For FCM analysis of bacteria, approximately 1.0 × 10^6^ CFU of an exponential-phase culture were transferred to 1-ml microcentrifuge tubes and centrifuged at 5,000 × *g* for 5 min. After removal of the supernatant, the cells were resuspended in 98 μl of staining buffer (SB; 0.1% bovine serum albumin [Merck] in PBS [pH 7.4; Merck]), to which 2 μl of antibody was added. After brief vortexing, the tubes were incubated on ice for 30 min. Cells were centrifuged at 5,000 × *g* for 5 min, and the pellet was resuspended in SB, to which was added 1 μl of SYBR Gold (Thermo Fisher Scientific) working stock (see below) and, in the case of staining with anti-*Enterococcus* or anti-*Bacillus* antibodies, 0.5 μl of Alexa Fluor 647 Plus-conjugated goat anti-rabbit secondary antibody (catalog no. A32733; Thermo Fisher Scientific). In all tests, the final volume was 100 μl, and incubation proceeded at 37°C in the dark for 15 min. SYBR Gold working stocks consisted of 1:100 dilutions of the reagent supplied as a 10,000× concentrate in dimethyl sulfoxide, aliquoted, and stored at –20°C until needed. After incubation with SYBR Gold and (where unconjugated primary antibody was used) secondary antibody, cells were centrifuged at 5,000 × *g* for 5 min, the supernatant removed, and the pellet resuspended in 300 μl of SB containing PI (Merck) at a concentration of 5 μg ml^−1^. The final suspension was then analyzed using FCM.

In samples where esterase activity of cells was being assessed, the stain CellTrace calcein violet AM (Thermo Fisher Scientific) was included in the second staining step, along with SYBR Gold and the secondary antibody. Then, 1 mM working solutions of calcein violet AM were prepared on the day of each experiment. It was found through titrations that a 1:100 dilution gave the best separation of positive and negative populations; higher concentrations appeared to reduce cell viability (data not shown).

Purified mouse IgG (Thermo Fisher Scientific, catalog no. 10400C), marketed as an isotype control and being essentially serum IgG against a multiplicity of targets, was used to test the binding of S. aureus strains to IgG antibodies against multiple targets. A final concentration of 30 μg ml^−1^ mouse IgG was used. Secondary antibodies to detect primary mouse IgG for these experiments were all used at a final concentration of 7 μg ml^−1^: Alexa Fluor 647-conjugated whole goat IgG anti-mouse (Jackson ImmunoResearch, Cambridgeshire, UK; catalog no. 115-605-164), Fab fragment goat anti-mouse IgG (Jackson ImmunoResearch, catalog no. 115-607-003), or F(ab′)_2_ fragment goat anti-mouse IgG (Jackson ImmunoResearch, catalog no. 115-606-062).

### Flow cytometry.

Unless otherwise stated, FCM was carried out using a FACSAria IIIu Sorter (Becton Dickinson, Wokingham, UK) fitted with a 488-nm (blue) 20-mW laser, a 633-nm (red) 18-mW laser, a 405-nm (violet) 50-mW laser, and a 561-nm (yellow-green) 20-mW laser. Quality control was carried out on a daily basis using cytometry setup and tracking beads (Becton Dickinson) and Sphero Rainbow calibration particle (8-peak) beads (Becton Dickinson). Acquisition was carried out using the instrument’s 70-μm nozzle at the default pressure. All fluorescence channels, as well as forward scatter (FSC) and side scatter (SCC), were set to logarithmic gain, and the signal area of each parameter was used (as opposed to height or width). Triggering was set to the blue laser, a window extension of 2 was used during acquisition, and thresholds were set using the logical combination “FSC or SSC.” The fluorescence of SYBR Gold (Thermo Fisher Scientific), a cell-permeant nucleic acid dye with an excitation maximum (λ_ex_) at 495 nm and an emission maximum (λ_em_) at 495, was captured using the blue laser’s 530/30 detector. The fluorescence of PI (Merck), a nucleic acid dye which only enters cells with permeable membranes, with a λ_ex_ in the visible spectrum at 577 nm (but which is excited at an efficiency of 40% at 488 nm) and an λ_em_ at 620 nm, was captured using the blue laser’s 610/20 detector. PE, which has an λ_ex_ at 565 nm and an λ_em_ at 576 nm, had its fluorescence captured using the yellow-green laser’s 582/15 nm detector. Calcein violet AM, a nonfluorescent permeant dye, is cleaved by intracellular esterases to the fluorescent product calcein violet, which has an λ_ex_ at 400 nm and an λ_em_ at 452 nm, and its fluorescence was captured using the violet laser’s 450/40 detector. Compensation between the fluorochromes was carried out using single-stained controls of the dyes. Acquisition proceeded at rates of between 5,000 and 10,000 events per second, with at least 10,000 events which fell into the “Live” cell gate being recorded. Version 8.0.1 (build 2014 07 03 11 47) of FACSDiva software (Becton Dickinson) was used for acquisition. Data were saved and exported in the FCS 3.0 format.

The nucleic acid signal from SYBR Gold was used to construct a “Cell” gate in a cytograph of SYBR Gold fluorescence versus FSC. A second gate in a cytograph of FSC versus SSC was used to remove any remaining debris and aggregates. A third gate in a plot of SYBR Gold versus PI fluorescence was used to distinguish “Live” from “Dead” cells. In experiments where calcein violet AM staining was also included, a plot of fluorescence versus PI was used to distinguish the following populations: “double negative,” “calcein violet positive,” “double positive,” and “PI positive.” A final gate of SYBR Gold versus PE fluorescence quantified the percentage of cells from each of the aforementioned gates to which the PE-conjugated mouse monoclonal anti-human CD44 antibody had adhered.

For experiments where violet laser excitation was not required and where the secondary anti-rabbit antibody was used, FCM was carried out using an Accuri C6 (Becton Dickinson), which has a 488-nm 50-mW solid state blue laser and a 640-nm 30-mW diode laser. SYBR Gold and thiazole orange (TO; Merck; a nucleic acid binding dye, λ_ex_ at 514 nm, λ_em_ at 531 nm) fluorescence was captured using the FL1 detector (530 ± 15 nm), PI using the FL3 detector (>670 nm), and Alexa Fluor 647 Plus fluorescence (λ_ex_ at 650 nm, λ_em_ at 665 nm) using the FL4 detector (675 ± 12.5 nm). Samples were run at the instrument’s low setting (corresponding to a flow rate of 14 μl min^−1^), with an FSC threshold of 2,000. A compensation of 21.5% was applied to remove the spillover of SYBR Gold into the FL3 channel. A total of 10,000 PI-negative events were acquired per sample. Cells from this PI-negative gate (presumed intact cells) were gated into the FL4 channel, where the secondary antibody’s signal was measured. Files were exported for analysis in the FCS 3.0 format.

### Binding of various *Staphylococcus* species to mouse anti-human antibody.

The extent of the following *Staphylococcus* species’ capacity to bind PE-conjugated mouse monoclonal anti-human CD44 antibody was initially tested, with two replicates per strain being stained: S. epidermidis, S. aureus NCTC 8325, S. aureus chicken ready-meal isolate, *S. hyicus* subsp. *hyicus* DSM 20459, S. lentus ATCC 738152, and *S. xylosus* ATCC 700404. The Gram-negative strain, E. coli ATCC 1175, was also stained with antibody to provide a nonspecific binding control using a species that does not express SpA.

### Effect of heat killing on antibody binding.

The signals from PE-conjugated mouse monoclonal anti-human CD44 antibody from untreated exponential-phase cultures versus heat-killed cells of S. epidermidis, the S. aureus chicken ready-meal isolate, *S. hyicus* subsp. *hyicus* DSM 20459, S. lentus ATCC 738152, and *S. xylosus* ATCC 700404 were compared, with two replicates per treatment receiving staining. Heat killing was achieved by exposing cultures to 95°C for 10 min and resulted in >99% of cells appearing in the PI-positive region when analyzed using FCM.

### Physiology of *Staphylococcus* strains stored in water or PBS.

Next, 1-ml portions of stationary-phase cultures of S. epidermidis, the S. aureus chicken ready-meal isolate, *S. hyicus* subsp. *hyicus* DSM 20459, S. lentus ATCC 738152, and *S. xylosus* ATCC 700404 were transferred to 1.5-ml microcentrifuge tubes and centrifuged at 5,000 × *g*; their supernatants were removed, and the pellets were suspended in sterile PBS (pH 7.4 [Merck]) or water. Suspensions were incubated at 4°C for 24 h before being stained with SYBR Gold, calcein violet AM, and PI and analyzed using FCM. One sample per treatment received staining.

### Influence of growth phase on antibody binding.

Approximately 1.0 × 10^6^ CFU of the S. aureus chicken ready-meal isolate, S. aureus NCTC 8325, *S. hyicus* subsp. *hyicus* DSM 20459, S. lentus ATCC 738152, and *S. xylosus* ATCC 700404 from lag, exponential, stationary, and death phases of cultures were stained with PE-conjugated mouse monoclonal anti-human CD44 antibody, SYBR Gold, calcein violet AM, and PI and analyzed using FCM. One sample per treatment received staining. The percentages of cells staining “double negative,” “calcein violet positive,” “double positive,” and “PI positive” in a plot of calcein violet versus PI fluorescence were recorded. Gating on each of these populations to a plot of SYBR Gold fluorescence versus PE fluorescence established the percentage of cells reacting with the anti-CD44 antibody.

### Influence of cell physiology on antibody binding.

Approximately 1.0 × 10^6^ CFU from exponential-phase cultures of the S. aureus chicken ready-meal isolate were subjected to the following treatments: 0.09% (wt/vol) potassium sorbate, 12% NaCl, 0.09% sodium citrate, 0.5% IGEPAL CO-630 (a nonionic, nondenaturing detergent; Merck), and 0.25% (wt/vol) thymol (Thermo Fisher Scientific)—all for 30 min at 37°C, followed by heat treatment at 50°C for 30 min. Three replicates per treatment received staining. After treatment, the cells were stained and analyzed as described above. The effects of these treatments on cell viability were also recorded using plate counting.

### Antibody binding of *Staphylococcus* species spread on stainless-steel surfaces.

Next, 1-ml portions of exponential-phase cultures of S. aureus NCTC 8325, the S. aureus chicken ready-meal isolate, *S. hyicus* subsp. *hyicus* DSM 20459, S. lentus ATCC 738152, and *S. xylosus* ATCC 700404 were spread over the surfaces of 12 × 12 cm food-grade stainless-steel coupons using sterile inoculation spreaders (Sarstedt). After air drying, wrapping in sterile aluminum foil, and incubation for 24 h at 37°C, the area inside a sterile template (20 cm^2^; Copan, Murrieta, CA) was swabbed using sterile viscose swabs bathed in Amies medium (Sarstedt). The resulting suspensions were stained as described above, and plate counting was performed (five replicates per species).

### Antibody binding by *S. aureus* in food systems.

Three foodstuffs (grated cheddar cheese, pasteurized whole milk, and coleslaw, all purchased from a local supermarket) were spiked with the S. aureus chicken ready-meal isolate to levels of approximately 1.0 × 10^6^ CFU g^−1^ (or ml^−1^ in the case of milk). Levels of S. aureus before and after spiking were first estimated by using plate counting on Baird Parker medium (Merck) and indicating background levels of S. aureus that were under the limit of detection prior to spiking. After overnight incubation at refrigeration temperature, spiked bacteria were then tested for their ability to bind to the PE-conjugated mouse monoclonal anti-human CD44 antibody. Four replicates per foodstuff received staining, except in the case of cheese, where two replicates were tested. In the case of the cheese and coleslaw samples, 5-g samples were aseptically weighed into 118-ml Whirl-Pak stomacher bags (Nasco, Fort Atkinson, WI); to these were added 50 ml of sterile water. Samples were then subjected to 2 min on the low setting of a model 400 Stomacher lab blender (Seward, Worthing, UK). Next, 1 ml of the resulting suspension was removed and centrifuged at 5,000 × *g* for 5 min, and the supernatant was removed. Then, staining with antibody, SYBR Gold, PI, and calcein violet AM was carried out as described above. In the case of milk, 1 ml was transferred to a 1.5-ml microcentrifuge tube and acidified by addition of 50 μl of 2 N HCl to coagulate caseins, followed by incubation at 37°C for 5 min with shaking at 300 rpm and centrifugation at 1,100 × *g* for 5 min. The supernatant was then transferred to a fresh 1.5-ml microcentrifuge tube and centrifuged at 5,000 × *g* for 5 min, and the pellet was stained as described above. Plate counting was also carried out on the foodstuffs, with 100 μl of the relevant dilutions plated out on bile esculin azide agar (Merck) and NA, and 1-ml aliquots were enumerated on MRS agar (de Man, Rogosa, and Sharpe; Merck) using the pour plate technique. Plate count data revealed that only in coleslaw was the natural microflora at a level similar to that of spiked S. aureus chicken ready-meal isolate (counts on NA showed that ∼1.1 × 10^6^ CFU g^−1^ were present).

### Binding of polyclonal antibodies raised against other species to *Staphylococcus* species.

Exponential-phase cultures of E. faecalis ATCC 19433, E. faecium ATCC 19434, S. epidermidis, S. aureus NCTC 8325, S. aureus mixed pepper isolate, *S. capitis* subsp. *capitis* ATCC 35661, *S. hyicus* subsp. *hyicus* DSM 20459, and *S. xylosus* ATCC 700404 were stained either with primary anti-*Enterococcus* antibody as described above, followed by staining with SYBR Gold, secondary antibody, and PI, or with secondary antibody alone, SYBR Gold, and PI. One sample per condition received staining, and these were analyzed using FCM, and their Alexa Fluor 647 Plus fluorescence levels were compared. Similarly, the above-mentioned *Staphylococcus* species were stained with one of the three anti-*Bacillus* antibodies or with secondary antibody only and, where the positive control was purified, spores of B. cereus NCTC 7464.

### Binding of purified mouse IgG to *S. aureus*.

To test the reaction of S. aureus strains to purified mouse IgG, 1/100 dilutions of overnight S. aureus NCTC 8325, DSM 1104, and DSM 20231 were centrifuged at 10,000 × *g* for 5 min, and the pellet was resuspended in PBS containing 0.05% Tween 20. Mouse IgG was added to the cells at 30 μg ml^−1^, followed by incubation at 37°C for 1 h. Cells were washed in PBS by centrifugation at 10,000 × *g* for 5 min. The pellet was resuspended in PBS-Tween 20 containing 7 μg ml^−1^ Alexa Fluor 647-conjugated whole anti-mouse IgG, Fab fragment goat anti-mouse IgG, or F(ab′)_2_ fragment goat anti-mouse IgG and then incubated at 37°C for 30 min. The sample was washed in PBS by centrifugation at 10,000 × *g* for 5 min and resuspended in PBS containing 1 μg ml^−1^ TO (Merck). After incubation at 37°C in the dark for 20 min at room temperature, the cells were analyzed by FCM. This experiment was repeated once.

### Blocking of *S. aureus* binding of polyclonal antibodies raised against other bacterial species.

Three commercial products were tested for their ability to block SpA sites on S. aureus NCTC 8325 and thereby prevent binding of polyclonal anti-*Enterococcus* polyclonal primary and anti-rabbit secondary antibodies to this species: normal rabbit serum (catalog no. 011-000-120; Jackson ImmunoResearch), purified mouse IgG, and FcR blocking reagent (catalog no. 130-059-901; Miltenyi Biotec, Bisley, UK). For the initial experiment, rabbit and mouse sera at dilutions of 1:20, 1:10, and 1:5 in a final volume of 100 μl were used, while FcR blocker was tested at dilutions of 1:10, 1:5, and 2:5. Blocking was carried out on ice for 15 min. After this experiment, trials concentrated on the use of FcR blocker only: rabbit serum eliminated all signal from the polyclonal antibody and produced an unreliable blocking of S. aureus binding to primary and secondary antibody, whereas mouse IgG only weakly diminished S. aureus antibody binding (data not shown). FcR blocker at a dilution of 2:5 was then tested against S. epidermidis and S. *hyicus* subsp. *hyicus* DSM 20459.

In order to test whether FcR blocker worked in another polyclonal antibody system, S. aureus NCTC 8325, S. epidermidis, and *S. hyicus* subsp. *hyicus* DSM 20459 were exposed to a 2:5 dilution of the product for 15 min on ice before being stained with one of the three primary anti-*Bacillus* antibodies, followed by staining with secondary antibody. Samples were stained with secondary antibody only in order to assess the effect of FcR blocker on its nonspecific binding.

### Blocking of binding of purified mouse IgG to *S. aureus*.

The blocking of the nonspecific binding of S. aureus NCTC 8325, DSM 1104, and DSM 20231 to mouse IgG was attempted by incubating the cells with goat IgG (Thermo Fisher Scientific; catalog no. 31245), a product marketed as an isotype control but which is a mixture purified serum IgG of many antibodies of different specificities, at concentrations of 0, 0.11, 0.22, and 0.44 mg ml^−1^ for 30 min at 37°C prior to primary mouse and/or secondary anti-mouse antibody incubations.

### Data analysis and presentation.

FCM 3.0 files were analyzed using FCSExpress Lite standalone research version 6.06.0014, 32-bit (DeNovo Software, Glendale, CA). Cytograms and FCM histogram overlays for publication were also prepared using this package. For all other data analysis and graph preparation, Microsoft Excel for Office 365, 64 bit (16.0.10730.20344; Microsoft Corporation, Redmond, WA), was used.
